# Actions Speak Louder Than Words: Sentiment and Topic Analysis of COVID-19 Vaccination on Twitter and Vaccine Uptake

**DOI:** 10.2196/37775

**Published:** 2022-09-15

**Authors:** Murooj Yousef, Timo Dietrich, Sharyn Rundle-Thiele

**Affiliations:** 1 Social Marketing @ Griffith Department of Marketing Griffith University Nathan Australia

**Keywords:** COVID-19, COVID-19 vaccination, sentiment analysis, public health campaigns, vaccine uptake, Twitter, social media, vaccines

## Abstract

**Background:**

The lack of trust in vaccines is a major contributor to vaccine hesitancy. To overcome vaccine hesitancy for the COVID-19 vaccine, the Australian government launched multiple public health campaigns to encourage vaccine uptake. This sentiment analysis examines the effect of public health campaigns and COVID-19–related events on sentiment and vaccine uptake.

**Objective:**

This study aims to examine the relationship between sentiment and COVID-19 vaccine uptake and government actions that impacted public sentiment about the vaccine.

**Methods:**

Using machine learning methods, we collected 137,523 publicly available English language tweets published in Australia between February and October 2021 that contained COVID-19 vaccine–related keywords. Machine learning methods were used to extract topics and sentiments relating to COVID-19 vaccination. The relationship between public vaccination sentiment on Twitter and vaccine uptake was examined.

**Results:**

The majority of collected tweets expressed negative (n=91,052, 66%) rather than positive (n=21,686, 16%) or neutral (n=24,785, 18%) sentiments. Topics discussed within the study time frame included the role of the government in the vaccination rollout, availability and accessibility of the vaccine, and vaccine efficacy. There was a significant positive correlation between negative sentiment and the number of vaccine doses administered daily (r_267_=.15, *P*<.05), with positive sentiment showing the inverse effect. Public health campaigns, lockdowns, and antivaccination protests were associated with increased negative sentiment, while vaccination mandates had no significant effect on sentiment.

**Conclusions:**

The study findings demonstrate that negative sentiment was more prevalent on Twitter during the Australian vaccination rollout but vaccine uptake remained high. Australians expressed anger at the slow rollout and limited availability of the vaccine during the study period. Public health campaigns, lockdowns, and antivaccination rallies increased negative sentiment. In contrast, news of increased vaccine availability for the public and government acquisition of more doses were key government actions that reduced negative sentiment. These findings can be used to inform government communication planning.

## Introduction

### Background

Vaccination is a widely debated topic, and vaccine hesitancy is a key barrier to national and international immunization efforts; research indicates that certain factors may overcome vaccine hesitancy and influence public sentiment [1]. Vaccine hesitancy is defined as the “delay in acceptance or refusal of vaccination despite the availability of vaccination services” [2]. The lack of trust in vaccines is a major contributor to vaccine hesitancy. Lee et al [3] found that vaccine-hesitant groups are the least likely to express COVID-19 vaccination willingness. Misinformation surrounding vaccine efficacy and side effects can result in the public losing trust in any vaccine, and these negative sentiments can reduce vaccine uptake [4].

Research evidence suggests that vaccine uptake reflects the dynamic interplay between information exchange through interpersonal communications, exposure to public health communication through the media [5], and observations and experiences with government actions (or lack thereof). Scholars have identified social media as an important channel for observing and understanding the public’s thoughts and feelings [6]. People use social media to share their thoughts and beliefs, including views on public health efforts, such as vaccination rollouts, enabling the understanding of public opinions on a large scale.

Public sentiment is defined as people’s opinions, sentiments, evaluations, attitudes, and emotions about a topic [7]. Regarding COVID-19 vaccination, public sentiment could relate to the government vaccination rollout and trust in the vaccine itself. This study investigates key events, such as the launch of public health campaigns, state lockdowns, antivaccination rallies, news related to vaccine efficacy, and vaccine mandates, to identify which government actions can change public vaccination sentiment in Australia.

### Social Media

Social media platforms allow people to express their opinions and emotions about different topics, including health care–related behaviors, such as vaccination. Research suggests that social media is critical in people’s vaccination decision-making process [8]. In terms of willingness to be vaccinated, social media can be a positive or negative influence. Although social media provides governments and health authorities with a platform to disseminate credible public health information that encourages vaccination [9], the same platform can fuel controversial vaccine debates, negatively influencing public opinions and sentiment about vaccines [8]. Previous research has shown that 30%-60% of information about vaccines on social media is antivaccine content [10]. The spread of antivaccination content has serious consequences, including an increase in vaccine hesitancy and delays in vaccine uptake [11,12]. The COVID-19 pandemic presented a contemporary setting to examine vaccine hesitancy, with the proliferation of antivaccination content hindering vaccine acceptance and negatively affecting changes in the vaccine uptake and vaccination sentiment [13,14]. Vaccine hesitancy is not unique to the vaccine for SARS-CoV-2. Vaccine hesitancy is also evident for other vaccine-preventable diseases, such as measles, mumps, and pertussis, and vaccine hesitancy has been linked to increased disease resurgence. The recent increasing number of deaths related to influenza and viral pneumonia warrants the examination of social media content to determine how it influences vaccine acceptance and uptake [8,15].

Social media is a highly volatile platform where people express positive and negative opinions. Hence, content relating to the COVID-19 vaccine differs daily, reflecting people’s actions and reactions [16]. Different elements may contribute to the change in opinions and sentiment about vaccines, such as national and global events (eg, vaccine trials), legislation (eg, mandating vaccinations), public health campaigns, and vaccine-related news reports [5,8,13]. For example, Tavoschi et al [8] analyzed tweets from 2016 to 2017 to assess the overall sentiments surrounding vaccines in Italy. They found that laws mandating vaccinations for children had a significant negative influence, while disease outbreaks significantly positively influenced public sentiment toward vaccination. Hence, studying the sentiment and opinions of the public during vaccination rollout periods is a good approach for determining vaccination rollout success or failure. Examining public sentiment can also help identify key drivers of positive and negative sentiments toward vaccines, enabling governments and communication agencies to understand which government actions improve the public’s sentiment toward vaccines. This study focuses on the Australian government’s effort to vaccinate the Australian population against COVID-19.

### COVID-19 Vaccination Rollout in Australia

Australia’s strategy to combat COVID-19 was to invest in vaccinating the population against SARS-CoV-2. An effective, safe, accessible, and available vaccine is considered an effective long-term solution to the COVID-19 pandemic. For this study, we defined accessibility as how easily a person could access an appointment for vaccination and availability as the number of available vaccine doses within the country. A critical step in this solution is to vaccinate a high proportion of the population while combating obstacles, such as misinformation, vaccine hesitancy, and lack of trust in government and scientific efforts. To date, only 1 study has analyzed Australian social media content on Twitter from January to October 2020. Kwok et al [17] found that there was general public support for infection control measures (eg, lockdowns) and an overall positive sentiment surrounding the vaccine highlighted by positive emotions, such as trust and anticipation. After that report was published, there were major developments in the vaccination rollout, new communication campaigns were launched, and government announcements were made in Australia. Therefore, additional research considering social media sentiment analysis is warranted.

### Timeline of Key Events During the Australian Vaccination Rollout

The Australian vaccination rollout started in early 2021, with the Australian health minister announcing the federal government’s goal of vaccinating all adult citizens by the end of October 2021 [18]. In May 2021, this goal was reset for the end of 2021 based on the available supply of approved vaccines. The Therapeutic Goods Administration approved 4 vaccines for Australian use in 2021: the Pfizer-BioNTech vaccine on January 25, the Oxford-AstraZeneca vaccine on February 16, the Janssen vaccine on June 25, and the Moderna vaccine on August 9. The first doses of the COVID-19 vaccine were administered on February 21, 2021, to high-priority groups (ie, front-line workers, health care workers, aged care residents, and workers). This first group received the Pfizer-BioNTech vaccine in highly televised settings to increase trust in the vaccine [19]. On March 22, vaccination of the second-highest-priority groups commenced, focusing vaccination efforts on adults aged >70 years, Aboriginal and Torres Strait Islander people aged >55 years, adults with underlying medical conditions, and emergency service workers. On May 3, adults aged >50 years became eligible for the vaccine, and thousands of participating general practitioners were permitted to administer vaccines in their clinics to expand the scale of the vaccination rollout. As new evidence emerged, suggested changes to the vaccination plan and rollout were observed. For example, the Australian Technical Advisory Group on Immunisation (ATAGI) advised the government to reserve the Pfizer-BioNTech vaccine for people <60 years of age and to administer the more widely available Oxford-AstraZeneca vaccine to adults aged ≥60 years of age. In July, as significant outbreaks of COVID-19 affected Australia’s most populated states (New South Wales [NSW] and Victoria), people within communities experiencing such outbreaks were advised to seek vaccination with any available vaccine. At that time, the ATAGI stated, “ATAGI reaffirms our previous advice that in a large outbreak, the benefits of the COVID-19 vaccine AstraZeneca are greater than the risk of rare side effects for all age groups” [20]. Residents aged 16-39 years became eligible to receive the Pfizer-BioNTech vaccine on August 30, and yet more venues were able to administer the vaccines, including vaccination hubs, pharmacies, and community centers. Finally, early adolescents aged 12-15 years became eligible to receive the Pfizer-BioNTech vaccine on September 3. As of October 12, 2021, Australia had administered 31,020,482 doses of COVID-19 vaccines across the country, with 82.8% of people aged 16 years and over having received at least 1 dose and 63.4% having received 2 doses [21]. Although the initial vaccination rollout in Australia was constantly criticized for its slow pace compared to other developed countries, by the end of 2021, vaccination rates in first-dose coverage had surged past many developed nations, including the United States and European Union nations [22]. As of March 2022, 95% of people in Australia over the age of 16 years had received at least 1 dose and over 94% of people had received 2 doses of a COVID-19 vaccine [23].

### Australian Public Health Communication Campaigns

Recent evidence has shown the role of public health campaigns in influencing vaccine-related perceptions and intentions during the COVID-19 pandemic [13]. During the rollout, Australia launched multiple health promotion campaigns aiming to encourage the uptake of vaccines as they became available to the public. The first few campaigns the Australian federal government launched followed a different strategy to the global effort, focusing on informative and data-driven messages rather than emotional and narrative strategies [24]. A few advertisements were launched informing the public of the availability of the vaccine to certain groups (eg, over 40-year-olds), featuring spokespeople from different health professions, such as Australia’s deputy chief medical officer. Such campaigns began in January 2021 and continued to air with multiple versions broadcast until July 2021. With an informational and scientific tone, these campaigns highlighted the safety of the vaccine and encouraged eligible people to register for vaccination. In July, the federal government published its plan to increase the uptake of COVID-19 vaccines through a national COVID-19 campaign, “Operation COVID Shield” [25]. With an AUS $2 billion (~US $1.4 billion) budget, the plan featured 3 main work streams: coordinate, motivate, and deliver. Under the motivate work stream, advertising campaign messaging aimed to “motivate eligible people in Australia to receive at least the first dose of the COVID-19 vaccine by 20 December 2021” [25]. Within this plan, 4 public health campaigns were scheduled to run between July and December, 2021. The 4 campaigns are listed in Multimedia Appendix 1.

To assess the effectiveness of these campaigns, the federal government continuously measured sentiment to monitor public confidence in the vaccination rollout [25]. The plan stated, “If...public sentiments are found to be declining, a communications plan will be developed and implemented” [25]. The government assessed sentiments through 2 main data sources, consumer surveys and insights on public sentiments from existing market research programs.

This study aims to assess the change in sentiment and uptake of the COVID-19 vaccine in Australia between February and October 2021. We extended previous research findings into the Australian vaccination rollout, analyzing the effect of public health communication campaigns and actions on vaccine-related sentiment and examining the effect of sentiment on vaccine uptake. Hence, the following research questions (RQs) were examined:

- RQ1. How did vaccination-related sentiment correlate to vaccination rates in Australia between February and October 2021?
- RQ2. How did certain events during the vaccination rollout in Australia relate to changes in the public sentiment toward COVID-19 vaccination?

## Methods

This study utilized machine learning tools to identify public sentiment and analyze emotions related to the vaccination rollout. The analysis of sentiment and vaccine uptake rates was conducted by 1-way ANOVA using SPSS Statistics v. 28 (IBM Corporation).

### Twitter

As 1 of the world’s biggest social network platforms, Twitter has 217 million active users, 5.8 million of whom are in Australia [26]. In recent years, Twitter has gained noticeable attention in the scientific literature [6,27]. Twitter users can share instant status updates (tweets) about a range of topics, including health-related content. Twitter is particularly useful as it shows real-time changes in public opinions, sentiments, and perceptions about vaccinations. Twitter is also an accurate, fast, and cost-effective data source compared to surveys and interviews [6,8,17]. Hence, Twitter was chosen as the data source for this study.

### Data Collection

Multiple methods are available to extract and analyze data from Twitter using artificial intelligence (AI) techniques. Practitioners and scholars are increasingly using social media listening platforms to extract social media data sets and analyze sentiments, topics, and public opinions [27]. Such tools help examine different RQs and inform marketing and communication strategies [28]. An important feature of such tools is the supply of social media data for specific topics, times, and locations. As this study required the collection of tweets and additional data, including date stamps, locations, and links to the tweets collected, the TrackMyHashtag tool was used. The tool provides a dashboard where general analysis of tweet volumes, locations, engagement, and impressions can be viewed and interacted with and the Twitter premium application programming interface (API) service accessed. TrackMyHashtag was used to collect COVID-19 vaccine–related tweets posted between February 1 and October 27, 2021. Following the methodology of Kwok et al [17], retweets, non-English tweets, and tweets with geolocations outside Australia were excluded. Search terms included “vacc OR vax OR vaccine OR vaccination” AND “covid or corona.” Boolean operators were used to ensure that tweets related to “vaccine” and “covid” were collected.

### Sentiment Analysis

Sentiment analysis is defined as “the application of natural language processing, computational linguistics and text analytics to identify and extract subjective information in source materials” [29]. Text analysis involves “information retrieval (IR), lexical analysis to study word frequency distributions, pattern recognition, tagging/annotation, information extraction, data mining techniques including link and association analysis, visualization and predictive analytics” [28]. An AI-based tool, BytesView, was used through Python to analyze the sentiment of tweets collected for this study [30]. The BytesView tool uses API keys to authenticate requests, enabling access to the tool via multiple applications. The BytesView Python client library was used to integrate the BytesView API into our Python application, enabling the sentiment analysis process. Tweets were rated based on 3 sentiments (positive, negative, and neutral). Further, the R library package *syuzhet* (R Foundation for Statistical Computing) [31], which applies Stanford’s CoreNLP on text against an emotion dictionary, was used to identify specific emotions within tweets (ie, anger, fear, anticipation, trust, surprise, sadness, joy, and disgust) [32]. The valence of a tweet was identified, and then the emotion recognized in the tweet was highlighted. Tweets were scored to reflect the sentiment and emotion within the text.

### Sentiment and Uptake Correlations

Vaccine uptake data are readily available from the Australian Department of Health website. Data from February to October 2021 were exported and listed per day. Pearson correlation coefficient testing (95% CIs) was undertaken to determine relationships between sentiment variables and vaccine uptake. Analysis was undertaken using mean daily, weekly, and monthly values for variables of interest.

### Key Influencing Events

Following the research of Tavoschi et al [8], a set of preselected vaccine-related events (eg, lockdowns) were identified and analyzed to determine their influence on vaccine-related sentiment and uptake. The events that the research team identified included the launch of public health campaigns, lockdowns, COVID-19 vaccine mandates in certain sectors, and changes in travel restrictions. The mean sentiment values from the 5 days preceding (pre-event), during, and after (postevent) each event were analyzed using 1-way ANOVA to assess the variation in sentiment related to these key events. Tukey post hoc testing was undertaken to determine differences within grouping variables across pre-, during-, and postevent time points. All data were analyzed using SPSS Statistics v. 28.0.0.0, and the level of statistical significance was set at *P*<.05.

### Topic Modeling

Several Python3 libraries, including Pandas, Regex, Re, and Numpy, were used to develop the latent dirichlet allocation (LDA) topic model. Nltk and spaCy were used for natural language processing, Gensim LDA multicore was used for topic modeling, and PyLDAvis was used for data visualization. The Gensim LDA model has been previously used for topical analysis research [33] and was recently used to analyze COVID-19–related tweets in South Africa [34]. LDA topic modeling was conducted 4 times, first for the whole data set and then for each sentiment group. The model presents 10 topics for each group of tweets and defines the most frequently used words. We evaluated each model based on coherence and perplexity scores. Coherence is the measure of how interpretable topics are for humans, while perplexity measures the efficacy of generative models by measuring the probability of a topic being produced by the model on a data set. A low perplexity score indicates that the model can accurately predict the text corpora of interest [35].

The topic modeling analysis process had multiple steps. First, the data set was loaded into the Pandas data frame via the CSV reader, which extracted the content and sentiment classification from each tweet. Second, we removed all emoji characters and Universal Resource Locators (URLs) from each tweet. Third, stop words were extracted from the nltk library, and additional stop words were added by the research team (see Multimedia Appendix 2).

Before running the LDA model, all vocabularies were tokenized with 500 batches for each run; tokens were converted into strings, lemmatized, and then converted back into a string. Subsequently, a word dictionary was created from lemma tokens while filtering extreme values in all tokens. Once a corpus object from lemma tokens was created, the base of the LDA model was initiated using the LdaMulticore algorithm in Python. Model parameters were optimized based on perplexity and coherence scores. Topics were generated, and the final model perplexity and coherence scores were computed. To visualize the results, the model was plotted using the pyLDAvis library. All analysis steps were performed for each sentiment category (ie, all tweets, positive tweets, negative tweets, and neutral tweets).

### Ethical Considerations

The Institutional Review Board of Griffith University approved this study (2019/697).

## Results

This study collected 137,523 tweets published in Australia between February and October 2021 and analyzed these tweets based on topics, sentiments, and specific emotions.

### Topics

**Figure 1:**
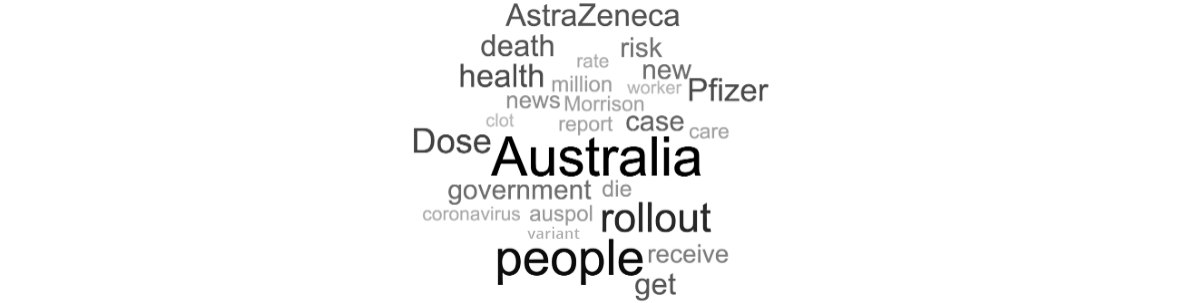
Word cloud of the most salient terms found in the total data set of COVID-19 vaccine–related tweets. Auspol: Australian politics.

**Figure 2:**
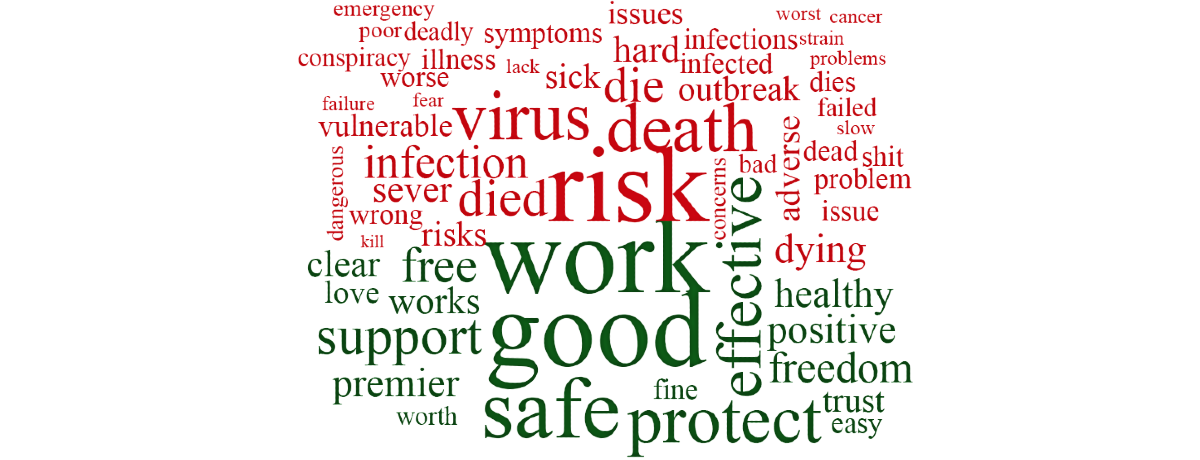
Word cloud based on the sentiment of COVID-19 vaccine–related tweets. Red words are representative of negative sentiment, and green words are representative of the positive sentiment toward COVID-19 vaccines.

In the overall data set, the top 10 most salient terms were “Australia,” “people,” “rollout,” “dose,” “health,” “Pfizer,” “AstraZeneca,” “death,” “case,” and “government.” Figure 1 shows an overall word cloud of the most frequent words found in the total data set of tweets. Figure 2 shows the most frequent words found in the tweets and their associated sentiment.

Topic modeling for all tweets and the 3 sentiment groups is presented separately. Additionally, the perplexity and coherence scores for each model are shown.

### All Tweets Within the Data Set

**Figure 3:**
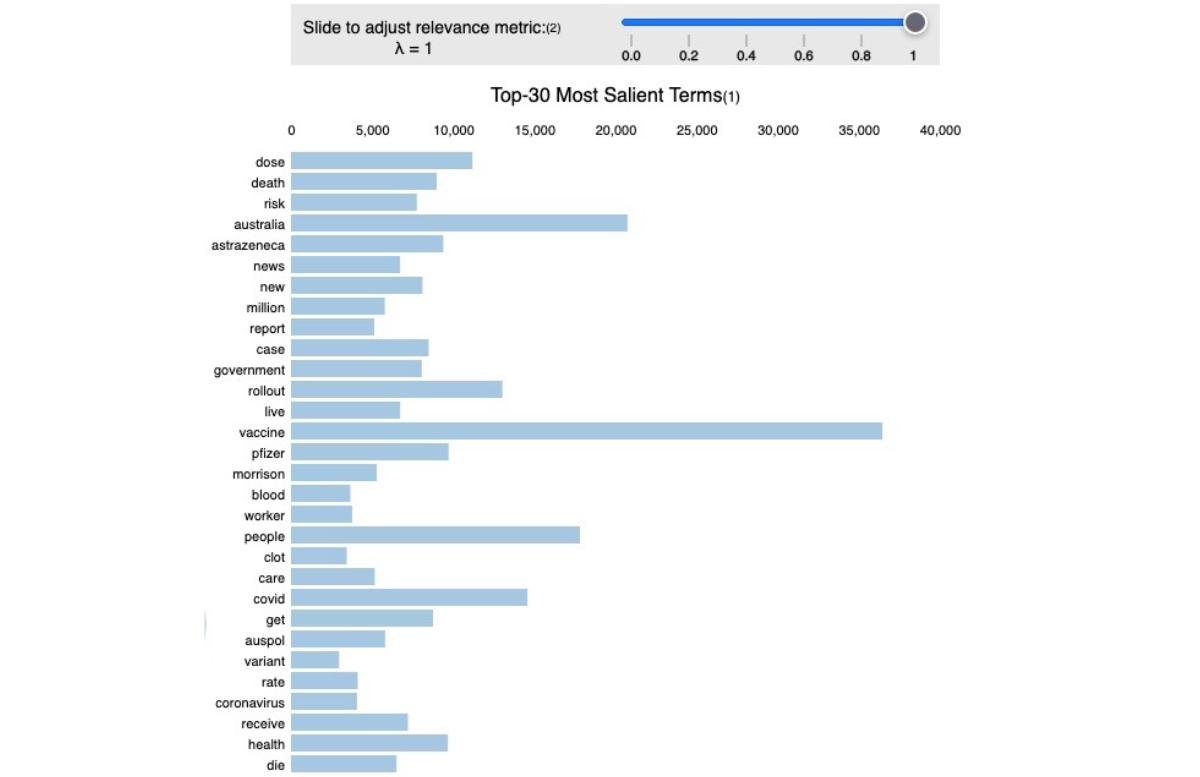
The 30 most salient terms in the entire data set of COVID-19 vaccine–related tweets.

When analyzing the topics within the whole data set of tweets, the model achieved a perplexity score of −8.46 and a coherence score of 48%, indicating that the topics were interpretable and definable. All model parameters are displayed in Table 1. The most common topics included news related to the vaccination rollout, vaccine effectiveness, and side effects; the government’s role in the vaccination rollout; outbreak prevention measures, such as lockdowns and border closures; vaccine availability and accessibility; and vaccine misinformation. The list of the extracted topics and their most common words is shown in Multimedia Appendix 3. The frequency of occurrence of the most salient words is shown in Figure 3.

**Table 1:** Topic modeling parameters.

Model parameters	All tweets	Positive	Negative	Neutral
Perplexity	−8.4613	−7.8601	−8.3145	−7.9399
Coherence	0.4859	0.4015	0.4584	0.4069
Number of topics	10	10	10	10
Random state	42	42	42	42
Chunk size	2000	2000	2000	2000
Passes	25	25	25	25
Decay	0.5	0.5	0.5	0.5
Iterations	100	100	100	100
Tweets, n (%)	137,523 (100)	21,686 (16)	91,052 (66)	24,785 (18)

#### Positive-Sentiment Tweets

**Figure 4:**
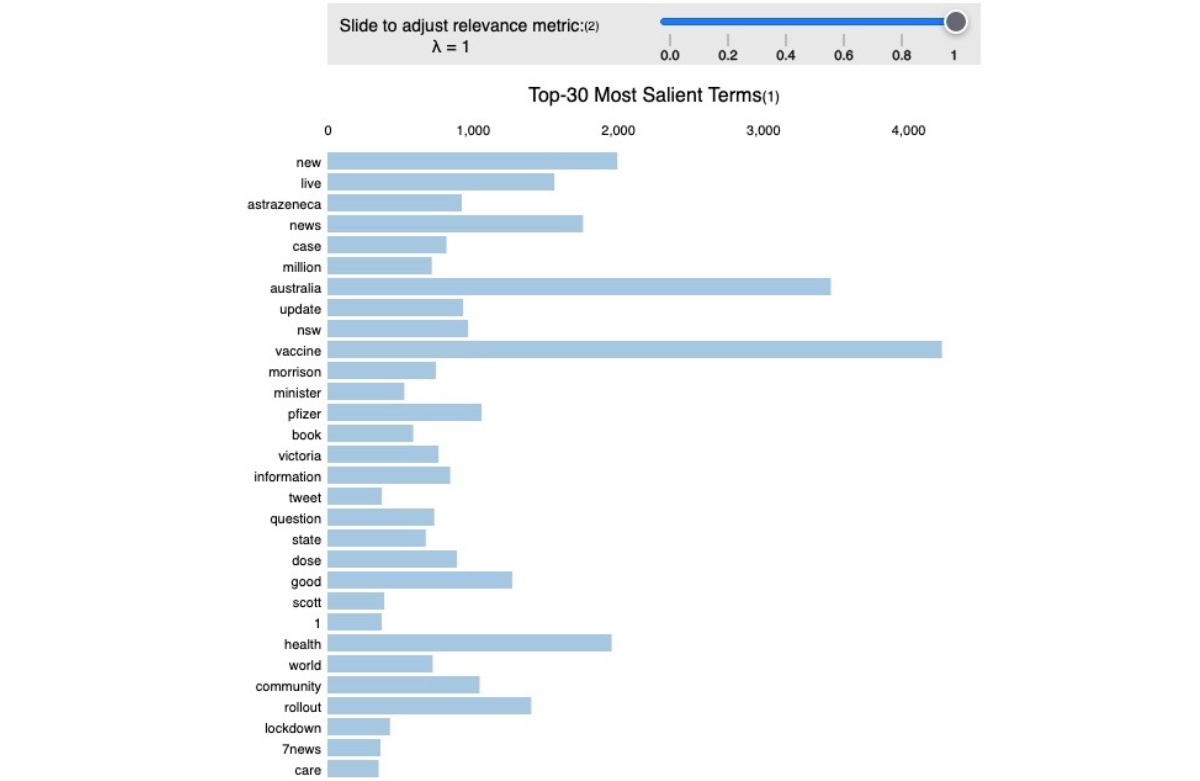
The 30 most salient terms in positive-sentiment COVID-19 vaccine–related tweets.

For this group of tweets, the model achieved a perplexity score of –7.86 and a coherence score of 40% (see Table 1). The list of the extracted topics is shown in Multimedia Appendix 4. People tweeted positively about being able to book a vaccination appointment, their appreciation for health workers, vaccine safety and related information, vaccine approvals, and vaccination rates. Figure 4 shows the frequency of words used within positive tweets in the data set.

#### Negative-Sentiment Tweets

**Figure 5:**
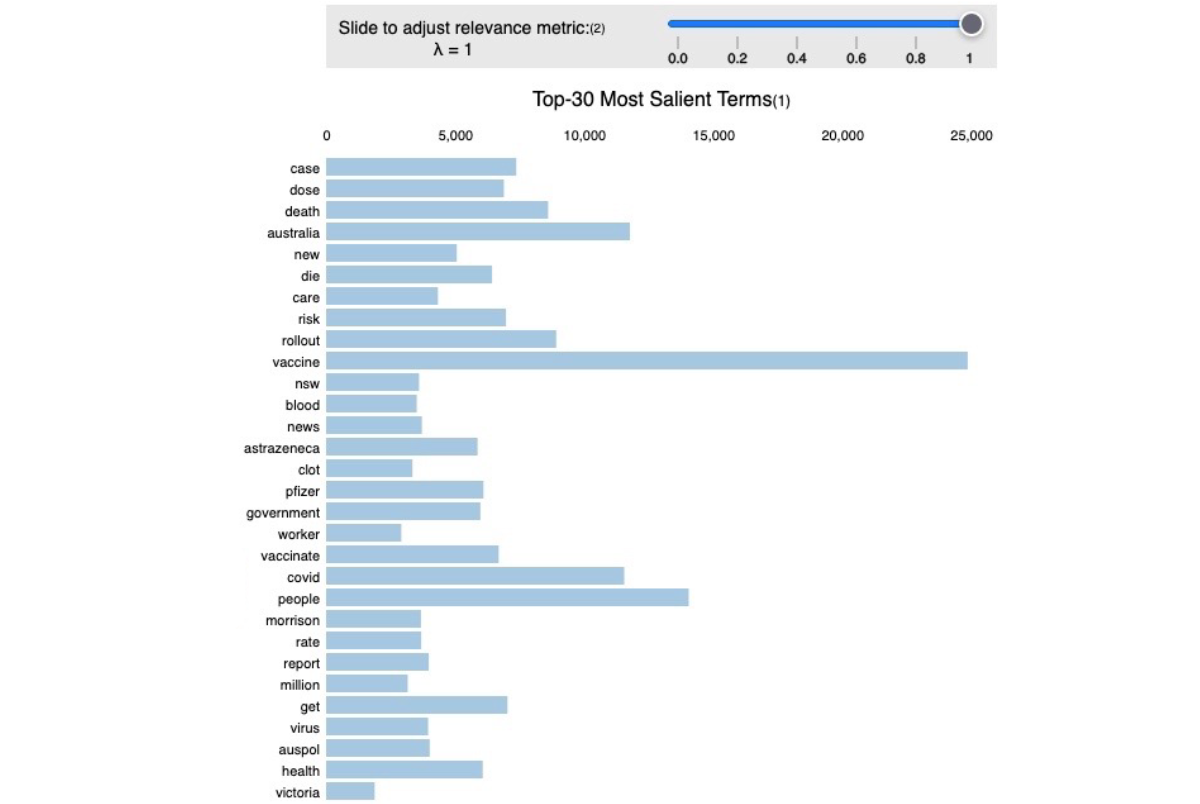
The 30 most salient words within negative-sentiment COVID-19 vaccine–related tweets.

For all negative tweets within the data set, the model achieved a perplexity score of –8.31 and a coherence score of 45% (see Table 1). Of the 10 identified topics, 5 (50%) were related to the government’s role in the vaccination rollout. These topics included discussions about vaccine accessibility, availability, approvals, and acquisition. Other topics included border closures, outbreak prevention measures, and vaccine safety and effectiveness. The list of the extracted topics is shown in Multimedia Appendix 5, and the most salient words within this group of tweets are shown in Figure 5.

#### Neutral-Sentiment Tweets

**Figure 6:**
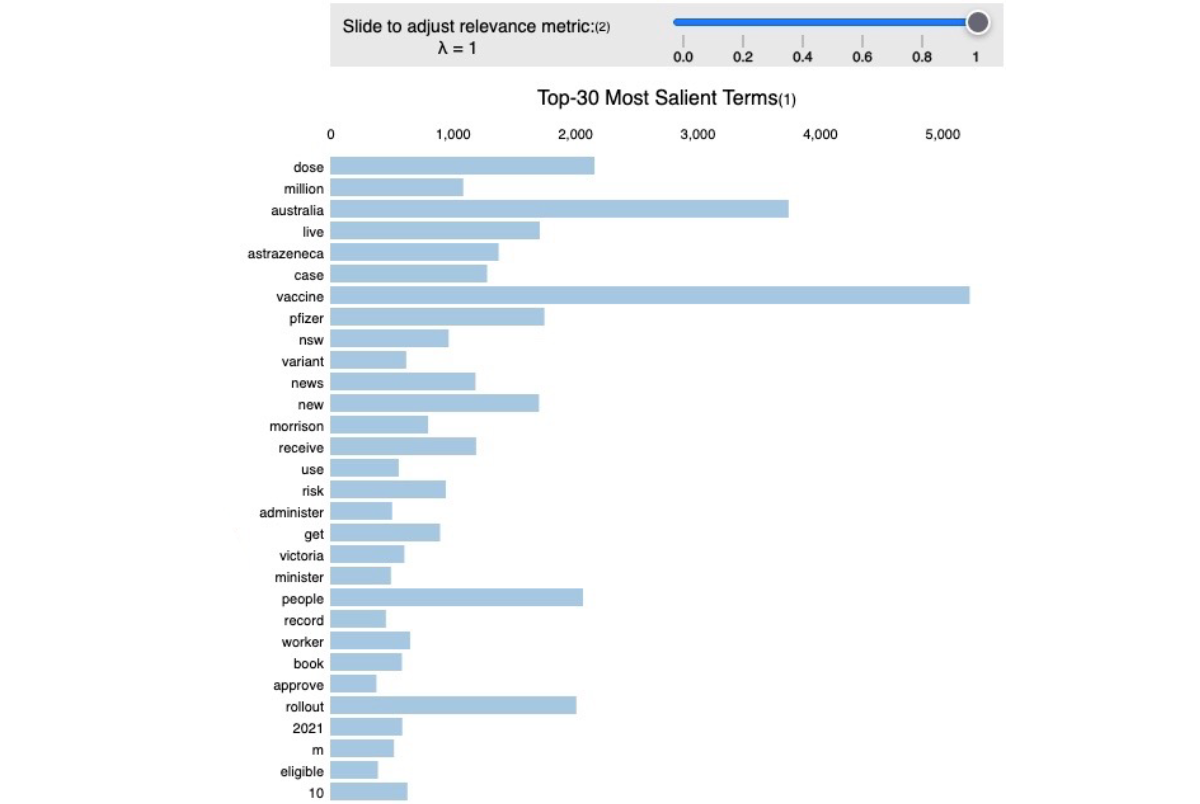
The 30 most salient words within neutral-sentiment COVID-19 vaccine–related tweets.

For tweets that showed no sentiment and were deemed neutral, the LDA model achieved a perplexity score of −7.93 and a coherence score of 40% (see Table 1). The list of extracted topics (see Multimedia Appendix 6) showed that tweets in this category discussed vaccine acquisition and accessibility news, vaccine mandates and work requirements, vaccine safety and efficacy, the role of the government in the vaccination rollout, risks of the vaccine, and vaccination approvals for children. The frequency of occurrence of the most salient words is shown in Figure 6.

### Sentiment

**Figure 7:**
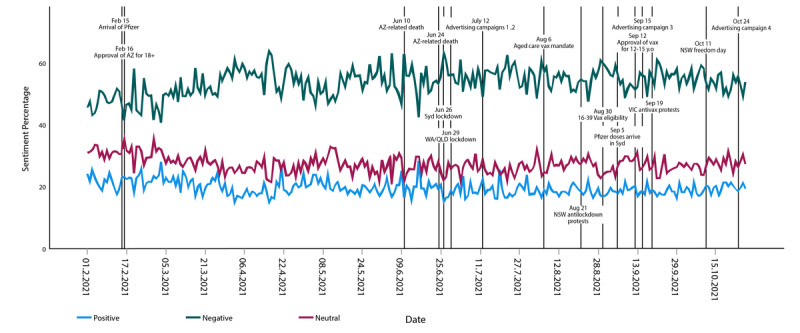
Key events and sentiments in COVID-19 vaccine–related tweets over time. NSW: New South Wales; QLD: Queensland; WA: Western Australia; Syd: Sydney; VIC: Victoria; AZ: AstraZeneca; y.o: years old.

**Figure 8:**
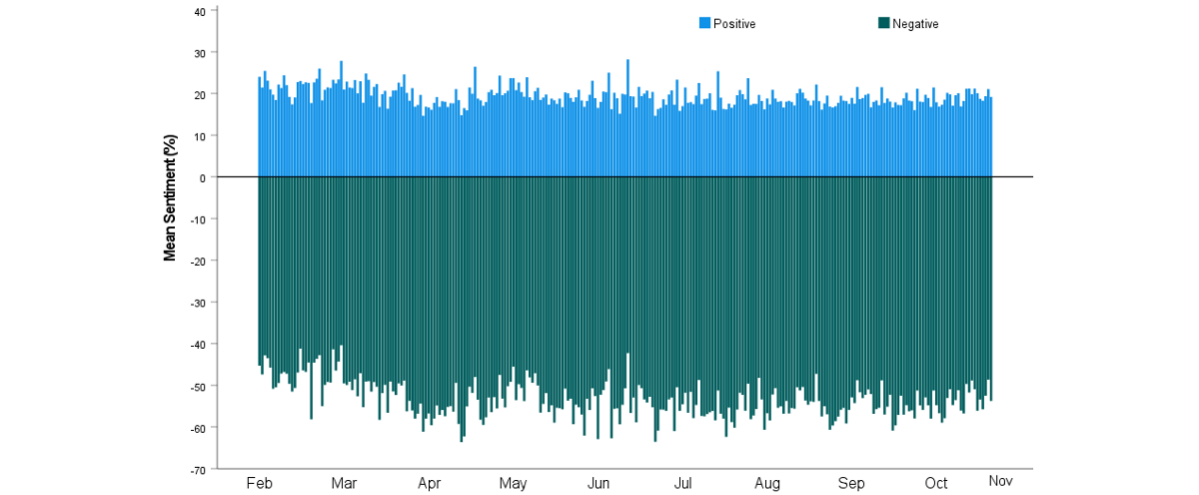
Positive and negative sentiments in COVID-19 vaccine–related tweets over time.

Most of the collected tweets expressed negative (n=91,052, 66%) rather than positive (n=21,686, 16%) or neutral (n=24,785, 18%) sentiments. Figure 7 shows the change in sentiment scores of all tweets between February and October 2021, along with the key events in this period. There can be a varying degree of positivity and negativity in each tweet; hence, the higher the sentiment score, the stronger the sentiment expressed in the tweet. Figure 8 shows the positive and negative sentiment levels across the 9 months of the study. Negative sentiment peaked on June 26, 2021, which aligned with the announcement of the greater Sydney lockdown in NSW, and dropped to a minimum on June 16, 2021, which aligned with the availability of the Oxford-AstraZeneca vaccine in selected pharmacies. Positive sentiment peaked on March 3, 2021, when it was announced that 250,000 doses of the Oxford-AstraZeneca vaccine were arriving from Italy. Positive sentiment dropped to its lowest point on April 16, 2021, after the vaccine-related death of a 48-year-old woman, 4 days after receiving the Oxford-AstraZeneca vaccine.

### Emotions

**Figure 9:**
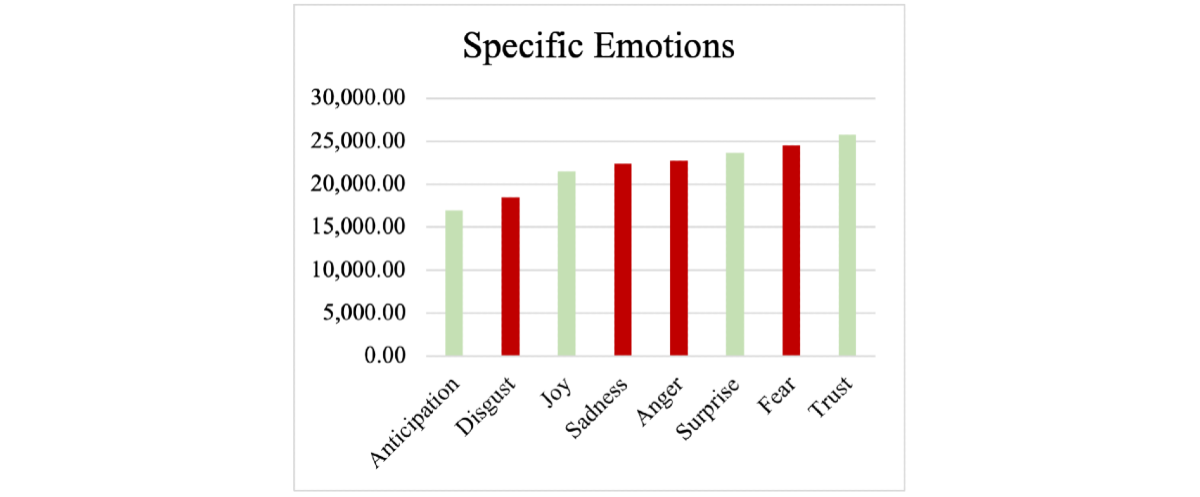
Emotions detected in COVID-19 vaccine–related tweets.

The emotions of fear, trust, surprise, and anger dominated the tweets collected for this study. The negative emotions expressed included anger (n=23,710, 16%), sadness (n=22,410, 15%), fear (n=21,540, 14%), and disgust (n=18,480, 12%). The most commonly expressed positive emotions were trust (n=25,730, 17%), anticipation (n=16,950, 11%), joy (n=12,300, 8%), and surprise (n=10,290, 15%). Figure 9 shows the frequency of each emotion in the tweets, with negative emotions in red and positive emotions in green.

### Uptake and Sentiment Over Time

**Figure 10:**
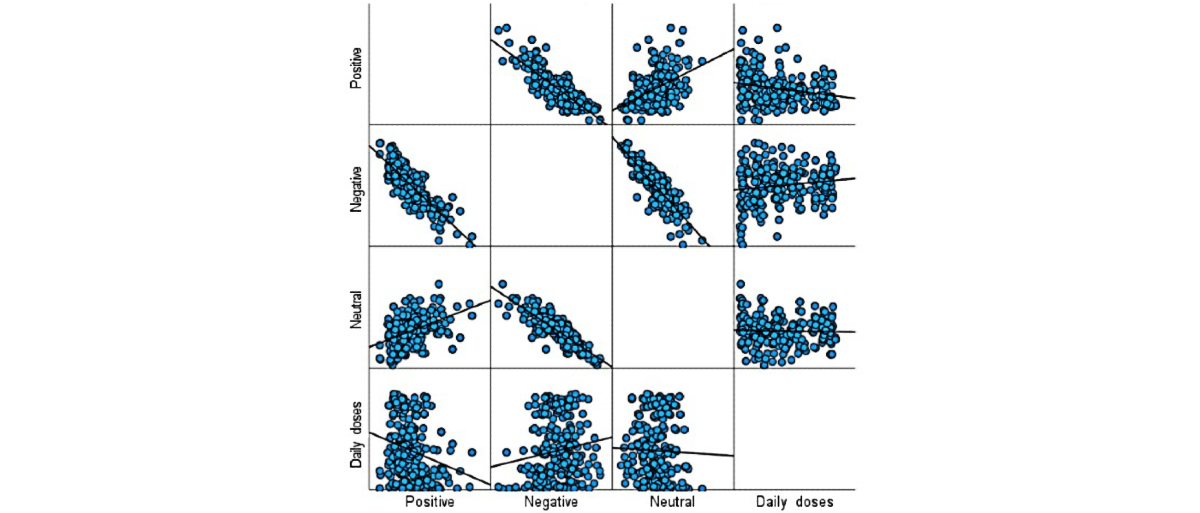
Scatter plot of correlation results between vaccine uptake and sentiments expressed in COVID-19 vaccine–related tweets.

When analyzing the relationship between sentiment and COVID-19 vaccine uptake, higher rates of negative sentiment were associated with increased vaccination uptake (see Table 2). Although people may have been expressing negative sentiments about the vaccination rollout, they were still getting vaccinated. A significant positive correlation between negative sentiment and daily doses administered was evident (r_267_=.15, *P*=.02). In contrast, when public sentiment was more positive, lower vaccine uptake rates were observed (r_267_=−.23, *P*<.001). Finally, when sentiment was neutral, there was no significant correlation in vaccine uptake rates (r_267_=−.03, *P*=.62). Figure 10 shows the correlation results as a scatter plot, identifying a weak correlation between sentiment and uptake.

**Table 2:** Daily sentiment and uptake correlation results.

	Positive	Negative	Neutral	Daily doses
Positive	1.00	N/A^a^	N/A	N/A
Negative	−0.84^b^	1.00	N/A	N/A
Neutral	0.48^b^	−0.88^b^	1.00	N/A
Daily doses	−0.23^b^	0.15^c^	−0.03	1.00

^a^N/A: not applicable.

^b^*P*<.01.

^c^*P*<.05.

### Key Events

The analysis by event was performed on a set of preselected events, including the launch of major national public health campaigns, announcements of vaccine availability, and the start and end of lockdowns. These key events are shown in relation to sentiment change in Figure 7.

#### Public Health Campaigns

**Figure 11:**
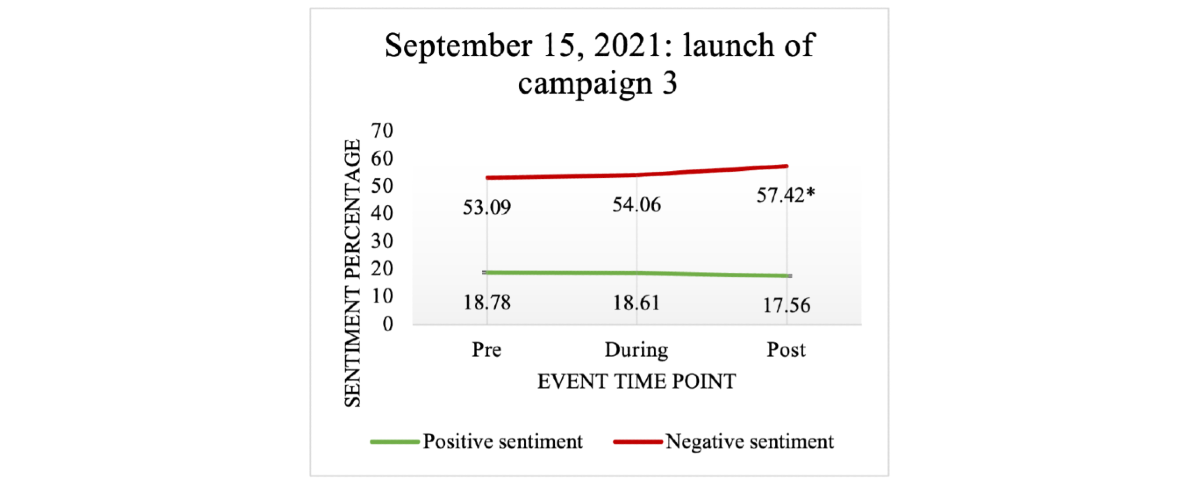
The effect of the September 15, 2021 “First Things First” vaccination campaign on negative and positive sentiments expressed in COVID-19 vaccine–related tweets.

The launch of the 2 first COVID-19 vaccine campaigns on July 12, 2021, had no significant effect on negative or positive sentiments expressed in tweets (see Table 3). However, Figure 11 shows that the third campaign, “First Things First,” which was launched on September 15, 2021, was associated with a significant increase in negative sentiment postevent compared to pre-event.

**Table 3:** Public health campaigns’ effect on public sentiment.

Event time point			July 12, 2021: campaigns 1 and 2				September 15, 2021: campaign 3		
			Mean (SD)	*P* value (compared to pre-event)		Mean (SD)			*P* value (compared to pre-event)
**Negative sentiment**									
	Pre-event	54.26 (32.48)		N/A^a^	53.09 (32.65)			N/A	
	During the event	54.91 (32.90)		.76	54.06 (32.83)			.52	
	Postevent	56.12 (32.41)		.11	57.42 (32.39)			<.001	
**Positive sentiment**									
	Pre-event	18.77 (22.44)		N/A	18.78 (22.55)			N/A	
	During the event	19.67 (23.39)		.34	18.61 (22.03)			.95	
	Postevent	18.53 (22.58)		.93	17.56 (21.39)			.12	

^a^N/A: not applicable.

#### Vaccine Acquisition– and Approval–Related Events

**Figure 12:**
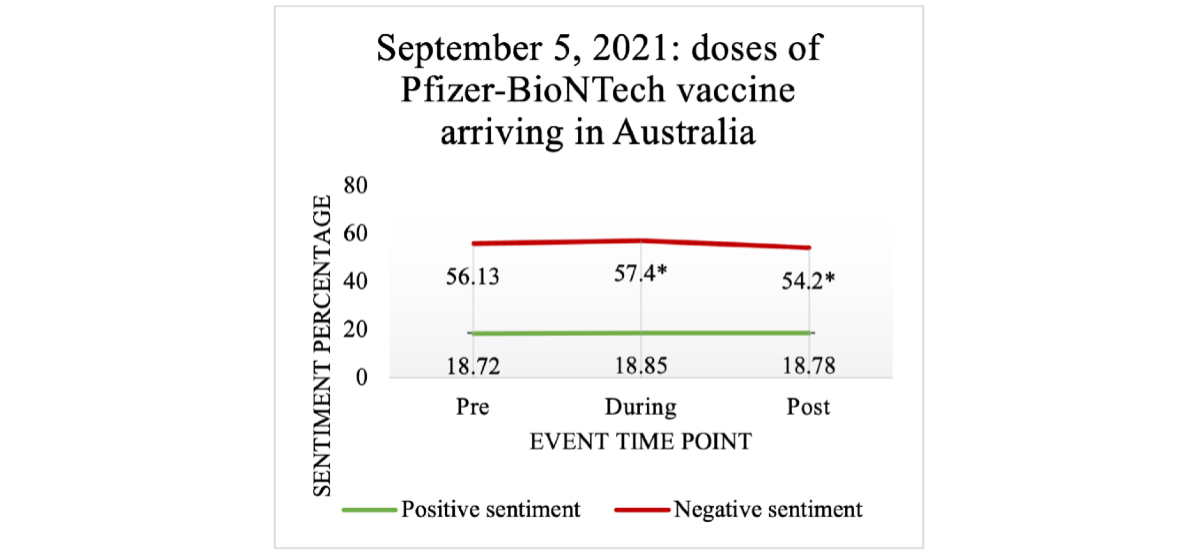
The effect of vaccine availability on negative and positive sentiments expressed in COVID-19 vaccine–related tweets.

The arrival of Pfizer-BioNTech vaccine doses in Australia on February 15, 2021, led to a significant increase in positive sentiment during the event compared to pre-event. The same event led to a significant decrease in negative sentiment during the event compared to pre-event. Similarly, the approval of the Oxford-AstraZeneca vaccine for people aged ≥18 years on February 16, 2021, led to a significant postevent increase in positive sentiment. This same event led to a significant postevent reduction in negative sentiment compared to pre-event. On September 5, 2021, the arrival of more doses of the Pfizer-BioNTech vaccine in Sydney resulted in a significant reduction in negative sentiment lasting for the 15 days of the event compared to pre-event, as shown in Figure 12. There was a significant decrease in negative sentiment when the Pfizer-BioNTech vaccine was approved on August 30, 2021, for people aged 16-39 years. However, when vaccination was approved on September 12, 2021, for people aged 12-15 years, there was a significant increase in negative sentiment compared to pre-event (see Tables 4-8).

**Table 4:** Vaccine acquisition and approval effect on public sentiment (February 15, 2021: arrival of Pfizer-BioNTech doses in Australia).

Event time point		Mean (SD)	*P* value (compared to pre-event)
**Negative sentiment**			
	Pre-event	49.19 (32.77)	N/A^a^
	During the event	45.04 (32.46)	<.001
	Postevent	47.99 (32.98)	.36
**Positive sentiment**			
	Pre-event	20.38 (23.68)	N/A
	During the event	22.69 (26.02)	.003
	Postevent	21.86 (25.06)	.07

^a^N/A: not applicable.

**Table 5:** Vaccine acquisition and approval effect on public sentiment (February 16, 2021: approval of Oxford-AstraZeneca for people aged ≥18 years).

Event time point		Mean (SD)	*P* value (compared to pre-event)
**Negative sentiment**			
	Pre-event	48.93 (32.77)	N/A^a^
	During the event	47.28 (32.63)	.16
	Postevent	46.86 (33.06)	.04
**Positive sentiment**			
	Pre-event	20.38 (23.88)	N/A
	During the event	21.65 (25.03)	.15
	Postevent	22.29 (25.54)	.01

^a^N/A: not applicable.

**Table 6:** Vaccine acquisition and approval effect on public sentiment (September 5, 2021: doses of Pfizer-BioNTech vaccine arriving in Sydney from the United Kingdom).

Event time point		Mean (SD)	*P* value (compared to pre-event)
**Negative sentiment**			
	Pre-event	56.13 (32.46)	N/A^a^
	During the event	57.40 (31.88)	<.001
	Postevent	54.20 (31.94)	.001
**Positive sentiment**			
	Pre-event	18.72 (22.36)	N/A
	During the event	18.85 (22.45)	.97
	Postevent	18.78 (22.55)	.99

^a^N/A: not applicable.

**Table 7:** Vaccine acquisition and approval effect on public sentiment (August 30, 2021: Pfizer-BioNTech vaccine approved for people aged 16-39 years).

Event time point		Mean (SD)	*P* value (compared to pre-event)
**Negative sentiment**			
	Pre-event	56.77 (32.19)	N/A^a^
	During the event	57.40 (31.88)	.76
	Postevent	54.20 (31.94)	.01
**Positive sentiment**			
	Pre-event	17.65 (21.26)	N/A
	During the event	17.83 (21.33)	.95
	Postevent	18.75 (22.15)	.17

^a^N/A: not applicable.

**Table 8:** Vaccine acquisition and approval effect on public sentiment (September 12, 2021: vaccinations approved for people aged 12-15 years).

Event time point		Mean (SD)	*P* value (compared to pre-event)
**Negative sentiment**			
	Pre-event	52.06 (32.11)	N/A^a^
	During the event	54.34 (32.48)	.04
	Postevent	54.82 (32.97)	.01
**Positive sentiment**			
	Pre-event	19.26 (22.87)	N/A
	During the event	18.11 (21.79)	.16
	Postevent	18.55 (21.94)	.48

^a^N/A: not applicable.

#### Vaccine Side Effects and Death Events

**Figure 13:**
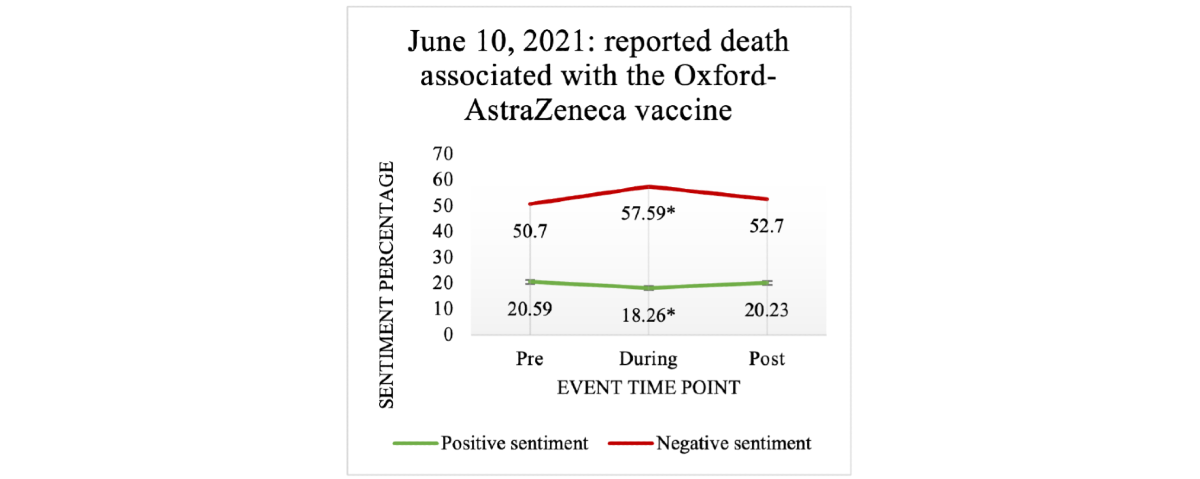
The effect of a vaccine-related death on negative and positive sentiments expressed in COVID-19 vaccine–related tweets.

On June 10, 2021, a death was attributed to the Oxford-AstraZeneca vaccine, resulting in a significant increase in negative sentiment during the event compared to pre-event, as shown in Figure 13. Similarly, positive sentiment significantly decreased during the event compared to pre-event. No significant effect was found on postevent sentiment. On June 24, 2021, another death was associated with the administration of the Oxford-AstraZeneca vaccine, resulting in similar trends in increased negativity and decreased positivity during the event compared to pre-event (see Table 9).

**Table 9:** Vaccine side effects’ and deaths’ influence on public sentiment.

Event time point		June 10, 2021: Oxford-AstraZeneca vaccine-related death					June 24, 2021: Oxford-AstraZeneca vaccine-related death		
		Mean (SD)		*P* value (compared to pre-event)		Mean (SD)			*P* value (compared to pre-event)
**Negative sentiment**									
	Pre-event	50.70 (33.36)	N/A^a^		53.21 (57.91)			N/A	
	During the event	57.59 (32.35)	<.001		57.91 (32.04)			<.001	
	Postevent	52.70 (33.71)	.10		55.90 (32.04)			.12	
**Positive sentiment**									
	Pre-event	20.59 (24.65)	N/A		19.60 (23.31)			N/A	
	During the event	18.26 (22.54)	.003		16.86 (20.06)			<.001	
	Postevent	20.23 (23.72)	.86		18.73 (22.18)			.34	

^a^N/A: not applicable.

#### Antivaccination and Antilockdown Rallies

**Figure 14:**
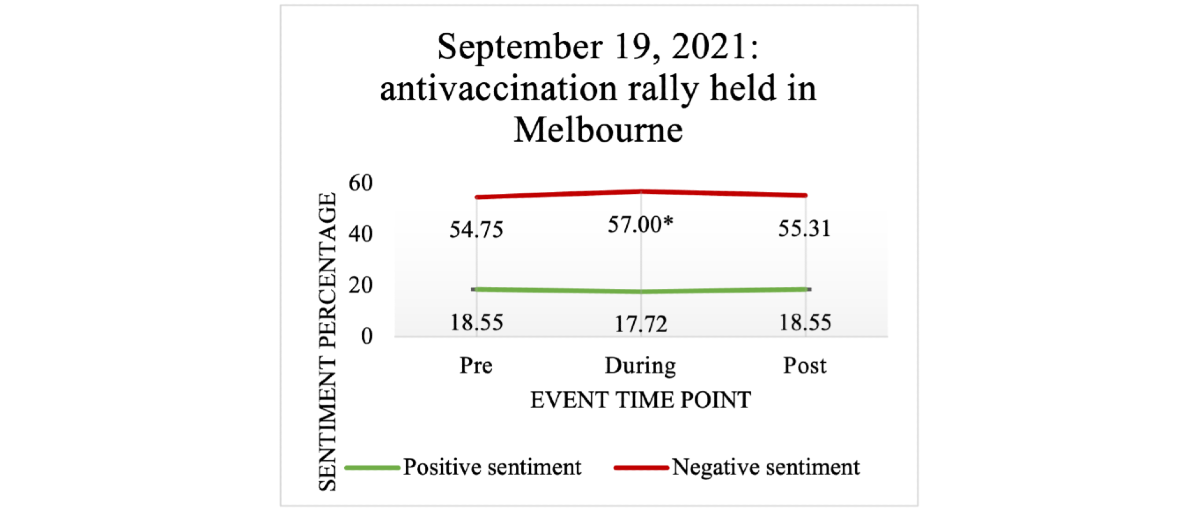
The effect of an antivaccination rally on negative and positive sentiments expressed in COVID-19 vaccine–related tweets.

Multiple antivaccination rallies were held across Australia in 2021. On September 19, 2021, a violent antivaccination rally occurred in Melbourne, Victoria—a city that had experienced the longest lockdown periods in the world—associated with a significant increase in negative sentiment during the event compared to pre-event, as shown in Figure 14. However, no significant effect on sentiment was found when comparing pre- and postevent negative sentiment or in comparisons of positive sentiment. Similarly, in NSW, when antilockdown and antivaccination rallies happened on August 21, 2021, negative sentiment significantly increased from pre- to postevent. Additionally, positive sentiment significantly decreased from pre- to postevent. No significant effect on either sentiment was found during the event (see Table 10).

**Table 10:** Antivaccination and antilockdown rallies’ effect on public sentiment.

Event time point		September 19, 2021: Melbourne antivaccination rally					August 21, 2021: NSW^a^ antilockdown and antivaccination rallies		
		Mean (SD)		*P* value (compared to pre-event)		Mean (SD)			*P* value (compared to pre-event)
**Negative sentiment**									
	Pre-event	54.75 (32.65)	N/A^b^		18.55 (22.20)			N/A	
	During the event	57.00 (32.55)	.04		17.72 (21.41)			.38	
	Postevent	55.31 (32.67)	.82		18.55 (22.14)			.99	
**Positive sentiment**									
	Pre-event	52.27 (32.50)	N/A		19.46 (23.13)			N/A	
	During the event	52.73 (32.58)	.87		18.80 (22.38)			.55	
	Postevent	57.95 (31.72)	<.001		17.35 (20.62)			.002	

^a^NSW: New South Wales.

^b^N/A: not applicable.

#### Lockdowns and Legislations

Lockdowns across different states had varying effects on sentiment. When a snap lockdown occurred in Queensland (QLD) and Western Australia (WA) on June 29, 2021, postevent negative sentiment significantly decreased compared to pre-event, indicating public support for the actions taken by these state governments. No significant effect on sentiments during the event was found. Positive sentiment also significantly increased throughout the events. In contrast, when a lockdown was announced for Greater Sydney on June 26, 2021, negative sentiment significantly increased during the 15 days of the event and postevent compared to pre-event, with the highest level of negative sentiment observed during the event. Positive sentiment significantly decreased during this lockdown period compared to pre-event. No significant effect on positive sentiment was found postevent compared to pre-event. When the lockdown was lifted in NSW on October 11, 2021, negative sentiment significantly decreased, but there was no significant effect on positive sentiment compared to pre-event (see Table 11).

**Figure 15:**
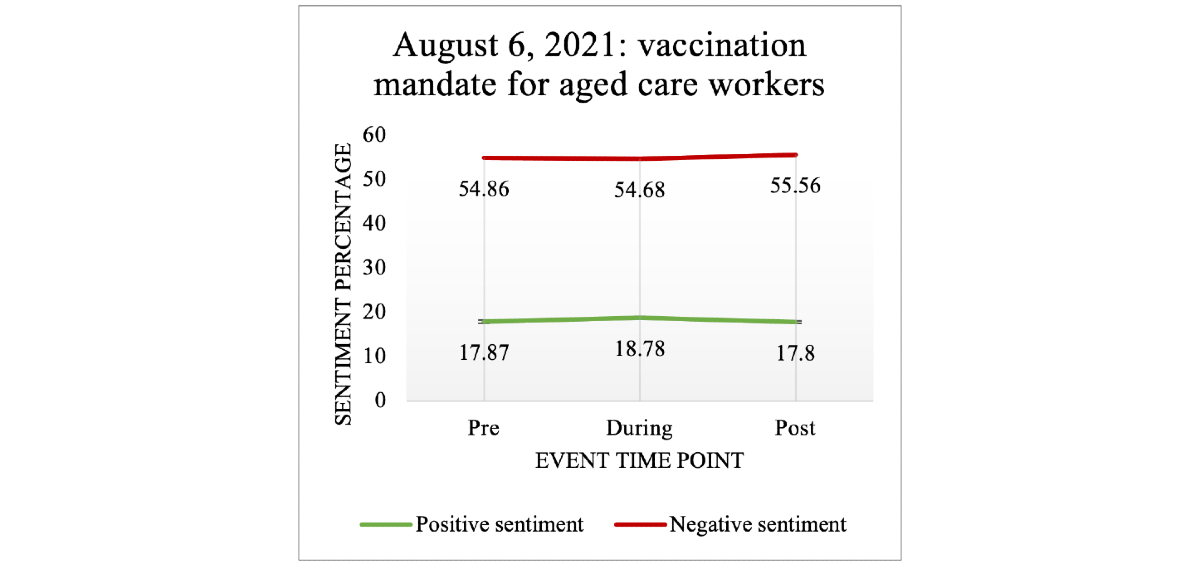
The effect of COVID-19 vaccination mandates for aged care workers on negative and positive sentiments expressed in COVID-19 vaccine–related tweets.

No significant changes in negative or positive sentiment were observed when COVID-19 vaccination was mandated for aged care workers on August 6, 2021 (see Table 12 and Figure 15).

**Table 11:** Lockdowns’ effect on public sentiment.

Event time point		June 29, 2021: QLD^a^ and WA^b^ lockdowns				June 26, 2021: Greater Sydney lockdown			October 11, 2021: Sydney lockdown lifted			
		Mean (SD)		*P* value (compared to pre-event)		Mean (SD)	*P* value (compared to pre-event)		Mean (SD)		*P* value (compared to pre-event)	
**Negative sentiment**												
	Pre-event	57.91 (32.04)	N/A^c^		52.67 (32.87)		N/A	55.90 (32.07)			N/A	
	During the event	55.90 (32.80)	.09		58.43 (31.63)		<.001	52.67 (32.74)			.001	
	Postevent	53.94 (32.64)	<.001		54.87 (33.12)		.05	52.42 (33.09)			<.001	
**Positive sentiment**												
	Pre-event	16.86 (20.06)	N/A		19.70 (23.37)		N/A	18.41 (22.02)			N/A	
	During the event	18.73 (22.18)	.01		16.67 (19.86)		<.001	19.34 (23.01)			.32	
	Postevent	19.02 (32.64)	.002		19.34 (23.05)		.83	19.45 (23.28)			.23	

^a^QLD: Queensland.

^b^WA: Western Australia.

^c^N/A: not applicable.

**Table 12:** Vaccination mandate’s (August 6, 2021: for aged care workers) effect on public sentiment.

Event time point		Mean (SD)	*P* value (compared to pre-event)
**Negative sentiment**			
	Pre-event	54.86 (32.60)	N/A^a^
	During the event	54.68 (32.60)	.98
	Postevent	55.56 (31.85)	.73
**Positive sentiment**			
	Pre-event	17.87 (21.79)	N/A
	During the event	18.78 (22.32)	.31
	Postevent	17.80 (21.39)	.99

^a^N/A: not applicable.

## Discussion

### Principal Findings

This study aimed to examine the relationship between public sentiment and COVID-19 vaccine uptake and between public sentiment and key actions and events. Key findings of this study included a negative correlation between sentiment and vaccine uptake and a significant change in sentiment observed for vaccine accessibility and availability events. Key topics within the analyzed data set showed that the population was discontented with the government’s effort in managing the vaccination rollout. This study contributes to our understanding of the role of public sentiment in vaccine uptake and overcomes the limitations of conventional survey methods [36]. Although big data and semantic analysis have been used for COVID-19 research, such analysis is mostly used to understand public discussions about the pandemic and related control measures [17] and public behavior, such as panic buying [37]. There remains a research gap in using machine learning methods to understand COVID-19 vaccination sentiment and actions beyond the response bias that typically occurs in a representative sample population [38]. Further, this study evaluated the effects of different Australian public health campaigns on public sentiment, improving our understanding of these campaigns’ effectiveness and providing recommendations for future campaign improvements. Similarly, implications of government efforts during a vaccination rollout were discussed, highlighting the need to align messages with the accessibility and availability of vaccines.

### Sentiment as an Indicator of Vaccine Uptake

Previously, research relied on self-reported measures to understand vaccine sentiment and the willingness to be vaccinated [36]. This study extended on previous research that applied machine learning methods to illuminate the limitations of self-reported measures. Although the literature has long discussed the importance of sentiment in vaccination rollouts, this study directly assessed the relationship between sentiment and vaccine uptake. Our findings indicate a weak and somewhat contradictory relationship between vaccine sentiment and vaccination uptake. Although previous research found that positive sentiment (ie, confidence in the vaccine) and uptake increased significantly over time [36], our findings differ in direction and strength; we found a weak inverse relationship between sentiment and vaccine uptake. Although people in Australia expressed negative sentiments in tweets, many still received vaccines. In contrast, higher rates of positive sentiment were associated with lower observed uptake rates. These associations might point to the role of accessibility and availability. Public sentiment was positive when vaccines were readily accessible; however, a lack of supply, or availability, led to an increase in negative sentiment. This may reflect times when members of the Australian public were unable to be vaccinated, despite their best efforts. Periods of neutral sentiment did not affect vaccine uptake rates. Hence, involving the public and helping them understand the impact of their decisions by emphasizing the importance of vaccination are crucial to ensure higher vaccine uptake rates.

To explain our findings, we must highlight the nature of the expressed sentiments about the vaccine rollout in the collected data; it is important to distinguish between negative sentiment toward the vaccine and negative sentiment toward government actions supporting or inhibiting the vaccination rollout. This is especially important in the Australian context, as the rollout faced many delays and obstacles.

#### Negative Sentiment Toward the Vaccine Rollout

This study demonstrated that vaccine uptake increased, while the general sentiment about the vaccination effort was negative. These findings reflect issues surrounding vaccine availability and accessibility. The topic modeling results highlighted that the most frequently discussed topics in tweets were related to the vaccination rollout, not the vaccine itself. In fact, the specific emotions highlighted within the data showed that many tweets from Australia expressed high levels of trust in the vaccine, indicating that most Australians were confident in the vaccine. Negative emotions, such as anger, observed in this study were largely attributed to the role of the government in the rollout rather than vaccine efficacy or safety concerns. A significant drop in negative sentiment and an increase in positive sentiment after news of newly acquired doses arriving in Australia was publicized is further evidence supporting the role of access and availability in public sentiment toward the vaccination rollout. This is also supported by the fact that negative sentiment only significantly increased during events related to vaccine deaths and side effects and returned to similar pre-event sentiment levels when the events had passed. This finding may be specific to the Australian context, given that high vaccine acceptance rates were recorded during the rollout [39]; however, the issue most commonly experienced by members of the public was the inability to receive the vaccine due to limited availability. These findings support those of previous studies, which showed that vaccine sentiment and acceptability are limited indicators of vaccine uptake but other factors (ie, vaccine availability, the ability to take time off work, and crowded vaccination schedules) more strongly influence an individual’s decision to be vaccinated [40].

Looking at the Australian context more closely, major shifts in vaccine acceptance and confidence were seen from 2020 to 2021. A study of COVID-19 vaccine hesitancy in 2020 found that 7% of the Australian population were hesitant about receiving a COVID-19 vaccine and a further 6% would resist the vaccine uptake [41]. However, the uptake rate during the 2021 rollout indicated lower proportions of vaccine hesitancy, with the majority of the population aged ≥12 years receiving 2 doses of a COVID-19 vaccine by December 30, 2021. Hence the role of vaccine hesitancy toward COVID-19 vaccines may not be as significant in Australia as in other countries. In fact, the vaccine acceptance rate reached 80% during outbreaks of the Delta strain, indicating that the Australian population views the vaccine as a solution for the COVID-19 pandemic [39]. From the start of the Australian COVID-19 vaccination rollout in February 2021, evidence suggested that the main factors affecting vaccine uptake were the availability and accessibility of the vaccine. However, the media continued highlighting vaccine hesitancy as a factor in vaccine uptake, without evidence of high hesitancy rates [39]. A broader examination of general vaccine acceptance (eg, how parents approve of vaccinations for children) shows that the critical elements for high vaccine uptake include vaccine availability and ease of access to vaccination, rather than underlying hesitancy factors [42]. Barriers to vaccination have long included crowded vaccination schedules, slow dissemination of doses, and limited public access to vaccines [43]. The government’s failure to clearly disclose supply and access issues has inflated discussions and public perceptions of vaccine hesitancy. Although there is no doubt that vaccine skeptics who refuse to accept and trust globally endorsed scientific evidence around vaccines, including COVID-19 vaccines, exist within the Australian population [3], many other factors influence vaccine uptake rates [39]. Different barriers may arise with different vaccination rollouts and different time points in each rollout. For example, when a new vaccine is created, logistics and approvals may be key barriers, as identified in this study. Other barriers may include poorly timed public health campaigns that have a negative effect on public sentiment. Clear and transparent reporting of all barriers to vaccine uptake, mapped to different vaccine rollouts and time points, would reduce misperceptions in our community.

### The Influence of Key Events on Sentiment

This study is the first to examine the effect of vaccine-related events on vaccine sentiment. Events tested within this study included the launch of 4 public health campaigns, lockdowns, antivaccination rallies, news related to vaccine efficacy, and vaccination mandates. Each event showed varying effects on vaccine sentiment.

#### Public Health Campaigns

The campaigns launched by the government to encourage the uptake of COVID-19 vaccines resulted in increased negative sentiment, with some events having a more significant influence on sentiment than others. When COVID-19 vaccine–related tweets were closely analyzed, a general frustration was observed within the period of each campaign launch. For example, Twitter users pointed out that the fear-based campaign, showing a young patient fighting for their life in an intensive care unit, was ill-timed because there were no approved vaccines for the age group represented in the campaign (30-40-year-olds) at that time. Multiple paper also criticized this particular campaign, highlighting that inciting fear is not the solution. In a paper evaluating the campaign’s fear appeal, Speight [44] explains that the limitation of this approach is that it neglects people’s “capability and opportunity to make the change.” Similarly, campaigns with a more positive emotional approach were also met with negative sentiment because many fully vaccinated Australians were still in lockdown, awaiting higher vaccination rates across their state. Hence, an increase in negative sentiment was found around the time these campaigns were launched. In terms of effectiveness, the campaigns did not lead to positive public sentiment, which was the main evaluation measure for the overall vaccine campaign [25]. Therefore, public health messages may have been more effective if they were released at more appropriate times, suggesting the millions of dollars invested in these campaigns would have been better placed in widening vaccine access and availability [45].

#### Vaccine Acquisition and Approval Related Events

All events related to increasing vaccine accessibility and availability resulted in significant decreases in negative sentiment and increases in positive sentiment, except when the Pfizer-BioNTech vaccine was approved for children aged 12-15 years old. Similarly, Tavoschi et al.[8] found that the approval of vaccines for children in Italy negatively impacted public sentiment. Excluding when vaccines were approved for children, an increase in positive sentiment was observed when the government increased access to vaccines. Taken together, this examination of different government actions may indicate that actions (eg, securing more vaccines, extending age groups that could be vaccinated) spoke far more loudly to the Australian population than words (eg, public health communication campaigns).

Vaccine efficacy and safety were questioned when cases of blood clots caused by the Oxford-AstraZeneca vaccine were identified in Australia; however, sentiment analysis showed that negative sentiments only increased significantly during these events. The publicized mixed messages and advice related to the Oxford-AstraZeneca vaccine also contributed to the rise in negative sentiment during this period. Danchin and Buttery [39] discussed the government’s mixed messaging when the ATAGI revised the approval for the Oxford-Astra-Zeneca vaccine for certain age groups. People struggled to understand the related risks and benefits, and even health professionals were confused about the approved administration of the Oxford-AstraZeneca vaccine. Public debates of the risks and relative benefits of the Oxford-AstraZeneca vaccine were prominent in this period as politicians and health care professionals took the debate to mainstream media and social media platforms [46]. This shows that vaccination rollouts should include clear and easy-to-understand messages to the public when changes in approvals and recommendations occur to eliminate any spread of misinformation and rumors [47]. This confirms that public knowledge also influences vaccine uptake [48].

#### Protests, Lockdowns, and Vaccination Mandates

Protests against COVID-19 public health measures started in different parts of the world in mid-2020 [49]. In Australia, the majority of such events were seen in 2021, when states went into lockdowns and vaccination mandates were established for certain sectors. Our findings indicate that antivaccination and antilockdown rallies increased negative public sentiment; however, vaccine uptake rates were still increasing. Similar to previous research findings, our data set shows debates between users on Twitter about the agenda behind these protests, the violence against state officials, and conspiracy theories. Our findings support those of Martin and Vanderslott [49], which describe a conflict between personal values (eg, liberal values) and the collective culture with regard to individual choices (eg, I choose not to get vaccinated). Expression of these values on social media led to an increase in negative sentiment, yet vaccination rates also increased. Lockdowns had different effects based on location. This is relevant to the Australian context, given that certain states endured longer lockdowns (ie, Victoria and NSW) than others (ie, South Australia, WA, and QLD). Lockdowns were associated with high uptake rates of the vaccines. Discussions within the collected tweets showed that tweets highlighted the danger of socializing in large crowds without masks prior to achieving the vaccination of a large proportion of the population. This may explain the high vaccination rates during these periods, where being vaccinated was associated with regaining some freedoms [49,50]. Finally, mandating vaccinations for sectors such as aged care did not significantly influence sentiment or uptake. This is similar to the findings of Lee et al [3], which demonstrated that different motivation measures and legislations failed to change the likelihood of vaccine uptake for certain groups. Mandating vaccination may not influence behavior when strong anti- or provaccination beliefs are present [3]. This finding may be explained by the nature of the aged care sector and vaccination requirements. Aged care workers in Australia must receive a set of vaccinations to work in the sector (eg, hepatitis A and B vaccination). Therefore, mandating COVID-19 vaccination for aged care workers did not significantly influence sentiment, as this population is familiar with such requirements.

### Limitations and Future Research

This study aimed to extend the understanding of the effect of public sentiment on vaccine uptake and the role of different government measures in changing public sentiment. Certain limitations apply and must be considered when interpreting these findings. First, the study’s scope is specific to the Australian vaccination rollout; therefore, the results may not be generalizable. Future research should investigate global sentiment during vaccination rollouts since 2020. Similarly, the data examined for this research are specific to 2021, the time frame of the collected data. Changes in sentiment and vaccine uptake before and after the collected data may be present, including more recent variant outbreaks, such as the Omicron variant outbreak in early 2022. Second, this study used the daily number of administered doses during the study period as a measure of vaccine uptake. However, it must be noted that other measures, such as clicks and traffic to vaccine-booking portals, views, reach, and engagement, were not analyzed and evaluated in the study, limiting our evaluation of public health campaign effectiveness. This can be overcome by future research acquiring clicks and engagement data from agencies running these campaigns [51,52]. Third, the large sample size used in the study may have reduced the impact of random errors, resulting in a higher probability of finding statistically significant results [53]. Fourth, a time delay may be present for shifts in sentiment and vaccination, as people may take time before taking action. Future research may test sentiment correlation with vaccinations using a distributed lag model [54,55]. Distributed lag models aim to associate outcome variables with time-dependent predictors (eg, getting vaccinated). Finally, we acknowledge that bots may contaminate data collected from social media platforms. Future research should implement a data-cleaning process to eliminate the effect of bot data and ensure data are representative of public opinion [56]. Future research may also use topic modeling to identify repeated text, which would help eliminate news headlines and bot-generated content.

### Conclusion

This study examined the role of public sentiment on COVID-19 vaccine uptake within Australia. The examination of different government actions indicated that actions such as securing more vaccines had a significant effect on alleviating negative sentiment and encouraging vaccine uptake. Our findings indicate that trust in the vaccine was high, contrary to trust in government rollout efforts. Hence, when negative sentiment was prevalent, vaccine uptake remained high. Future efforts for vaccination rollouts should focus on ensuring vaccines are available and accessible to the public through more coordinated supply chain logistics. Further, it is important to align message delivery with supply chain timings and regularly monitor public sentiment and vaccine uptake through objective data collection methods to eliminate biases.

## Appendix

Multimedia Appendix 1 Public health vaccine campaigns in Australia.

Multimedia Appendix 2 Stop words used in collecting Tweets from February to October 2021.

Multimedia Appendix 3 Topics extracted from all tweets within the data set.

Multimedia Appendix 4 Topics extracted from positive-sentiment tweets.

Multimedia Appendix 5 Topics extracted from negative-sentiment tweets.

Multimedia Appendix 6 Topics extracted from neutral-sentiment tweets.

## Abbreviations

AI: artificial intelligence
API: application programming interface
ATAGI: Australian Technical Advisory Group on Immunisation
LDA: latent dirichlet allocation
NSW: New South Wales
QLD: Queensland
RQ: research question
WA: Western Australia
