# Supplementary material for: Actions Speak Louder Than Words: Sentiment and Topic Analysis of COVID-19 Vaccination on Twitter and Vaccine Uptake
Source: JMIR Form Res. 2022 Sep 15;6(9):e37775. doi: 10.2196/37775 (PMC9484485; doi:10.2196/37775)
Supplement: Multimedia Appendix 1 [file formative_v6i9e37775_app1.docx]

**Multimedia Appendix 1**

**Public health vaccine campaigns in Australia**

| Campaign | Launch date | Advertising messages | Visual |
| --- | --- | --- | --- |
| Motivate campaign 1 | July 12, 2021 | The “Arm yourself” campaign showed a diverse group of people who had gotten their vaccine. | 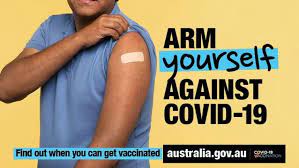 |
| Motivate campaign 2 | July 12, 2021 | A fear-based approach was used with imagery of a young patient fighting for their life as they battle COVID-19. | 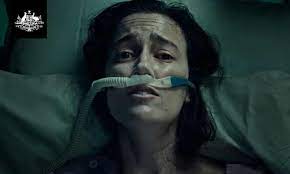 |
| Motivate campaign 3 | September 15, 2021 | A positive emotional appeal advertisement titled “First Things First,” this campaign showed images of freedom after achieving high vaccination rates. | 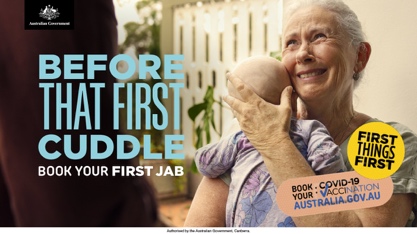 |
| Motivate campaign 4 | October 24, 2021 | The “Spread Freedom” campaign used images of life after high vaccination rates were achieved, with travel, social events, and concerts back to normal. | 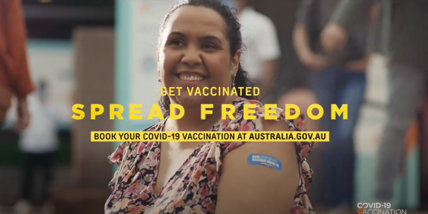 |
