# Supplementary material for: Actions Speak Louder Than Words: Sentiment and Topic Analysis of COVID-19 Vaccination on Twitter and Vaccine Uptake
Source: JMIR Form Res. 2022 Sep 15;6(9):e37775. doi: 10.2196/37775 (PMC9484485; doi:10.2196/37775)
Supplement: Multimedia Appendix 2 [file formative_v6i9e37775_app2.docx]

# Multimedia Appendix 2

**Stop words used in collecting Tweets from February to October 2021.**

[‘i’, ‘me’, ‘my’, ‘myself’, ‘we’, ‘our’, ‘ours’, ‘ourselves’, ‘you’, “you’re”, “you’ve”, “you’ll”, “you’d”, ‘your’, ‘yours’, ‘yourself’, ‘yourselves’, ‘he’, ‘him’, ‘his’, ‘himself’, ‘she’, “she’s”, ‘her’, ‘hers’, ‘herself’, ‘it’, “it’s”, ‘its’, ‘itself’, ‘they’, ‘them’, ‘their’, ‘theirs’, ‘themselves’, ‘what’, ‘which’, ‘who’, ‘whom’, ‘this’, ‘that’, “that’ll”, ‘these’, ‘those’, ‘am’, ‘is’, ‘are’, ‘was’, ‘were’, ‘be’, ‘been’, ‘being’, ‘have’, ‘has’, ‘had’, ‘having’, ‘do’, ‘does’, ‘did’, ‘doing’, ‘a’, ‘an’, ‘the’, ‘and’, ‘but’, ‘if’, ‘or’, ‘because’, ‘as’, ‘until’, ‘while’, ‘of’, ‘at’, ‘by’, ‘for’, ‘with’, ‘about’, ‘against’, ‘between’, ‘into’, ‘through’, ‘during’, ‘before’, ‘after’, ‘above’, ‘below’, ‘to’, ‘from’, ‘up’, ‘down’, ‘in’, ‘out’, ‘on’, ‘off’, ‘over’, ‘under’, ‘again’, ‘further’, ‘then’, ‘once’, ‘here’, ‘there’, ‘when’, ‘where’, ‘why’, ‘how’, ‘all’, ‘any’, ‘both’, ‘each’, ‘few’, ‘more’, ‘most’, ‘other’, ‘some’, ‘such’, ‘no’, ‘nor’, ‘not’, ‘only’, ‘own’, ‘same’, ‘so’, ‘than’, ‘too’, ‘very’, ‘s’, ‘t’, ‘can’, ‘will’, ‘just’, ‘don’, “don’t”, ‘should’, “should’ve”, ‘now’, ‘d’, ‘ll’, ‘m’, ‘o’, ‘re’, ‘ve’, ‘y’, ‘ain’, ‘aren’, “aren’t”, ‘couldn’, “couldn’t”, ‘didn’, “didn’t”, ‘doesn’, “doesn’t”, ‘hadn’, “hadn’t”, ‘hasn’, “hasn’t”, ‘haven’, “haven’t”, ‘isn’, “isn’t”, ‘ma’, ‘mightn’, “mightn’t”, ‘mustn’, “mustn’t”, ‘needn’, “needn’t”, ‘shan’, “shan’t”, ‘shouldn’, “shouldn’t”, ‘wasn’, “wasn’t”, ‘weren’, “weren’t”, ‘won’, “won’t”, ‘wouldn’, “wouldn’t”, ‘!’, ‘“‘, ‘#’, ‘$’, ‘%’, ‘&’, “‘“, ‘(‘, ‘)’, ‘*’, ‘+’, ‘,’, ‘-’, ‘.’, ‘/’, ‘:’, ‘;’, ‘<‘, ‘=‘, ‘>‘, ‘?’, ‘@’, ‘[‘, ‘\\’, ‘]’, ‘^’, ‘_’, ‘`’, ‘{‘, ‘|’, ‘}’, ‘~’, ‘covid19’, ‘c19’, ‘wax’, ‘vaxx’, ‘vax’, ‘vaccine’, ‘vaccination’, ‘covid-19’, ‘covid’, ‘vaccinate’, ‘pandemic’, ‘waxx’, ‘hi’, ‘\n’, ‘\n\n’, ‘&amp;’, ‘ ‘, ‘.’, ‘-’, ‘got’, “it’s”, ‘it’s’, “i’m”, ‘i’m’, ‘im’, ‘want’, ‘like’, ‘$’, ‘@’]
