# Supplementary material for: Actions Speak Louder Than Words: Sentiment and Topic Analysis of COVID-19 Vaccination on Twitter and Vaccine Uptake
Source: JMIR Form Res. 2022 Sep 15;6(9):e37775. doi: 10.2196/37775 (PMC9484485; doi:10.2196/37775)
Supplement: Multimedia Appendix 3 [file formative_v6i9e37775_app3.docx]

|  | Topic | Word cloud |
| --- | --- | --- |
| 1 | Australia Rollout News  australia news new live care worker rollout case coronavirus nsw | 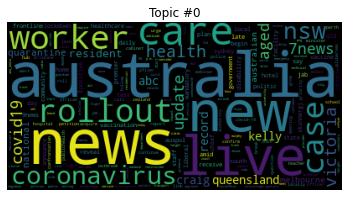 |
| 2 | Government Vaccination Rollout  rollout government Australia Morrison auspol scottmorrisonmp federal scott say minister | 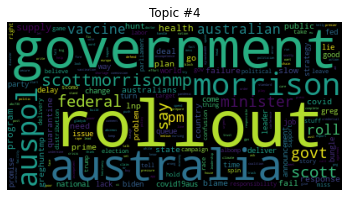 |
| 3 | Vaccine Effectiveness  vaccine variant effective new trial pfizer immunity study virus efficacy | 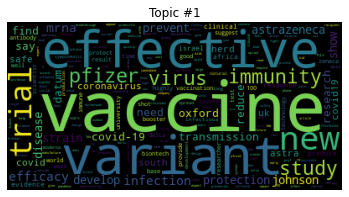 |
| 3 | Lockdowns and Border Closures  country rate australia lockdown covid high zero open need border | 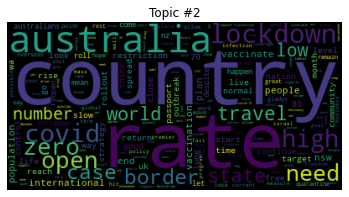 |
| 4 | Vaccine Death and Side Effects  death report case tga reaction number adverse datum link 2021 | 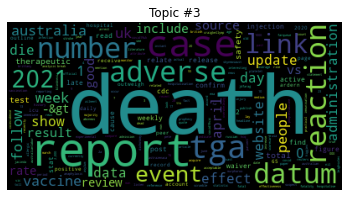 |
| 6 | Vaccine Perceptions  vaccine people covid get die know stop flu think need | 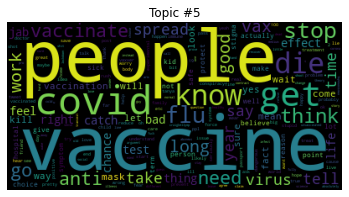 |
| 7 | Vaccine Misinformation  vaccine question use misinformation answer expert china medical health pay | 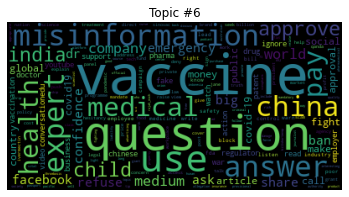 |
| 8 | Risks of AstraZeneca vaccine  risk astrazeneca vaccine blood clot az woman people health pfizer | 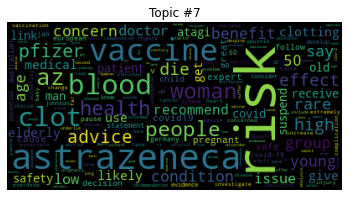 |
| 9 | Vaccine Doses Acquisition  dose million pfizer vaccine australia receive week year administer day | 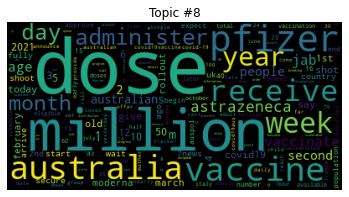 |
| 10 | Vaccine Accessibility  vaccine book today vaccination clinic health information gp community jab | 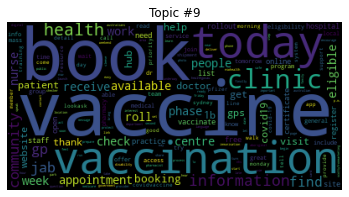 |
