# Supplementary material for: Actions Speak Louder Than Words: Sentiment and Topic Analysis of COVID-19 Vaccination on Twitter and Vaccine Uptake
Source: JMIR Form Res. 2022 Sep 15;6(9):e37775. doi: 10.2196/37775 (PMC9484485; doi:10.2196/37775)
Supplement: Multimedia Appendix 4 [file formative_v6i9e37775_app4.docx]

|  | Topic | Word Cloud |
| --- | --- | --- |
| 1 | Vaccine Accessibility  vaccine book question vaccination covid19 find thank answer health check | 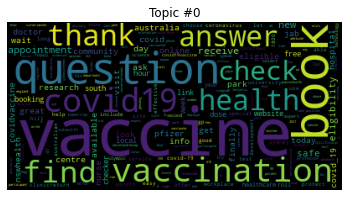 |
| 2 | Appreciation of Medical Staff  health receive today care community thank clinic staff worker group | 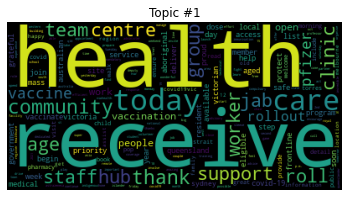 |
| 3 | Safety of Vaccine  good covid vaccine people know think great say Australia yes | 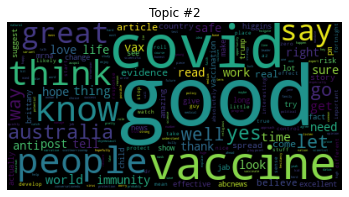 |
| 4 | Vaccine Information  vaccine information health people read vaccination risk covid19 rollout update | 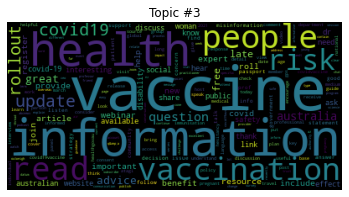 |
| 5 | Vaccine Approvals  astrazeneca australia news vaccine pfizer use johnson abc approve tga | 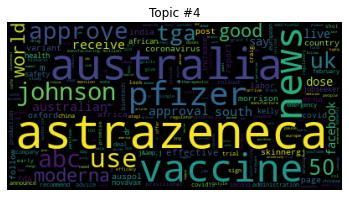 |
| 6 | Doses Administered in Australia  million tweet vaccine dose 1 5 3 2 australian 2021 | 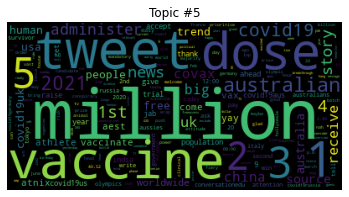 |
| 7 | Government Vaccination Rollout  minister australia rollout phase kelly health auspol scottmorrisonmp craig australian | 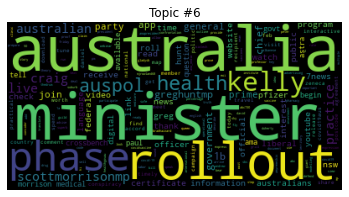 |
| 8 | National Target of Vaccinations  state community rollout government rate national vaccination people reach target | 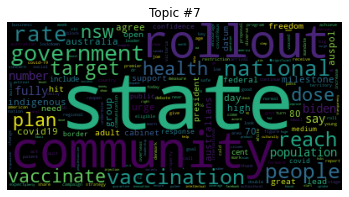 |
| 9 | Australia’s Vaccination Rate Compared to the World  new vaccine australia country world 7news jab covid19 daily government | 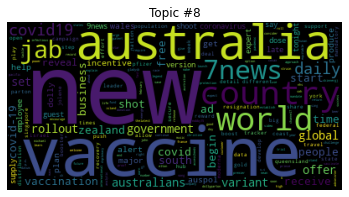 |
| 10 | COVID-19 Cases  live australia news case nsw update new morrison victoria coronavirus | 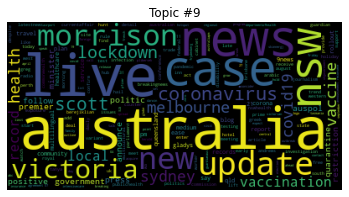 |
