# Supplementary material for: Actions Speak Louder Than Words: Sentiment and Topic Analysis of COVID-19 Vaccination on Twitter and Vaccine Uptake
Source: JMIR Form Res. 2022 Sep 15;6(9):e37775. doi: 10.2196/37775 (PMC9484485; doi:10.2196/37775)
Supplement: Multimedia Appendix 6 [file formative_v6i9e37775_app6.docx]

|  | **Topics** | Word Cloud |
| --- | --- | --- |
| **1** | Vaccine Acquisition  dose australia million pfizer receive vaccine administer 2021 rollout 10 | **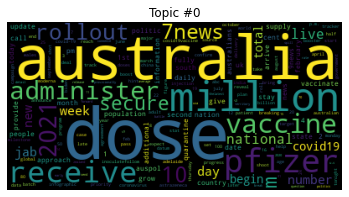** |
| **2** | Covid Vaccination Work Requirements  vaccine covid anti big know right think take test work | 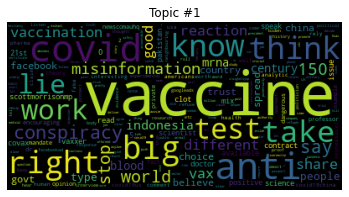 |
| **3** | Vaccination Accessibility  book health age eligible clinic receive phase live people available | **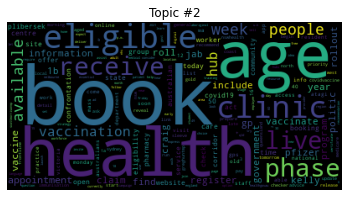** |
| **4** | Safety of Vaccine  vaccine people get good covid know today wait safe read | 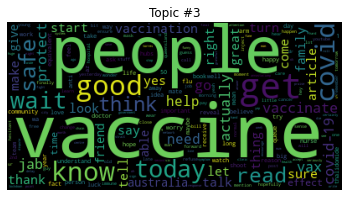 |
| **5** | Vaccine Effectiveness Evidence  vaccine variant astrazeneca expert effective new study oxford coronavirus say | **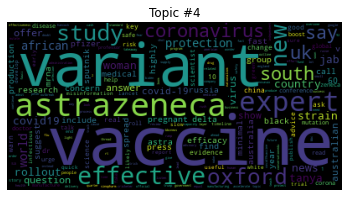** |
| **6** | Vaccination Rollout  people country rollout johnson vaccine know medium covid group need | 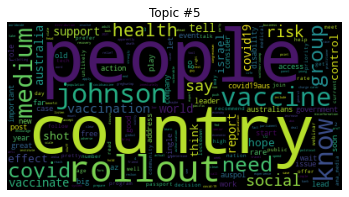 |
| **7** | Children Vaccination Approval  astrazeneca pfizer vaccine use approve trial australia child moderna tga | 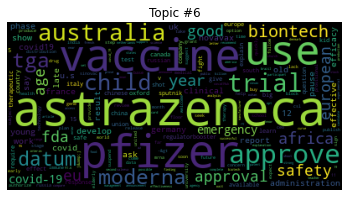 |
| **8** | Risks of Vaccine  risk covid rate vaccine people high long immunity need time | 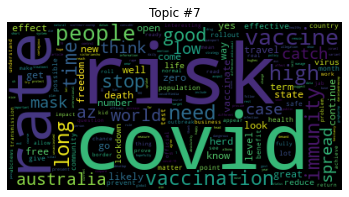 |
| **9** | Government Rollout Effort  australia government morrison health rollout auspol minister worker vaccine vaccination | 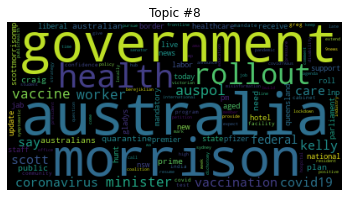 |
| **10** | COVID-19 Cases  case live australia new nsw news victoria record chief update | 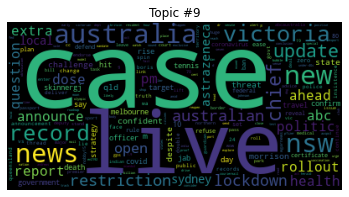 |
